# Gingival Response to Dental Implant: Comparison Study on the Effects of New Nanopored Laser-Treated vs. Traditional Healing Abutments

**DOI:** 10.3390/ijms21176056

**Published:** 2020-08-22

**Authors:** Barbara Ghinassi, Angela Di Baldassarre, Gianmaria D’Addazio, Tonino Traini, Mauro Andrisani, Giorgio Di Vincenzo, Giulia Gaggi, Maurizio Piattelli, Sergio Caputi, Bruna Sinjari

**Affiliations:** 1 Human Anatomy and Cell Differentation Lab, Department of Medicine and Aging Sciences, University “G.d’Annunzio” of Chieti-Pescara, 66100 Chieti, Italy; giulia.gaggi@unich.it; 2 Department of Medical, Oral and Biotechnological Sciences, University “G. d’Annunzio” of Chieti-Pescara, 66100 Chieti, Italy; gianmariad@gmail.com (G.D.); tonino.traini@unich.it (T.T.); mauro.andrisani@gmail.com (M.A.); maurizio.piattelli@unich.it (M.P.); sergio.caputi@unich.it (S.C.); bruna.sinjari@unich.it (B.S.); 3 Electron Microscopy Laboratory, University “G. d’Annunzio” of Chieti-Pescara, 66100 Chieti, Italy; 4 Department of Periodontics & Implant Dentistry, New York University, E 40th St #508, New York, NY 10016, USA; gtd2@nyu.edu

**Keywords:** soft tissue, dental implants, healing abutments, immunohistochemical analysis, coronal seal, ICAM-1, TNF-α, Cytokeratins, Desmoglein 3, IL-10

## Abstract

The health of peri-implant soft tissues is important for the long-term success rate of dental implants and the surface topography is pivotal in influencing it. Thus, the aim of this study was to evaluate, in human patients, the inflammatory mucosal microenvironment in the tissue surrounding a new, nanoscale, laser-treated healing abutment characterized by engineered nanopores versus a standard machined-surface. Analyses of anti- and pro-inflammatory markers, cytokeratins, desmosomal proteins and scanning electron microscopy were performed in 30 soft-tissue biopsies retrieved during second-stage surgery. The results demonstrate that the soft tissue surrounding the laser-treated surface was characterized by a lower grade of inflammation than the one facing the machined-surface, which, in turn, showed a disrupted epithelium and altered desmosomes. Moreover, higher adhesion of the epithelial cells on the laser-treated surface was detected compared to the machined one. In conclusion, the laser-treated surface topography seems to play an important role not only in cell adhesion, but also on the inflammatory makers’ expression of the soft tissue microenvironment. Thus, from a clinical point of view, the use of this kind of topography may be of crucial importance not only on healing abutments but also on prosthetic ones**.**

## 1. Introduction

Although dental implants have shown a cumulative survival rate of about 90% with a mean period of follow-up of 15.7 years [1], their biological and mechanical complications often remain unsolved [2,3]. In particular, one of the most important biological complications of the recent implantology is the onset of peri-implantitis, a destructive inflammatory lesion that affects both hard and soft tissues, causing bone loss and implant failure [4]. The resolution of inflammation during the inflammatory phase of wound healing, together with soft tissue attachment, is essential to avoid peri-implantitis and achieve long-term implant success [5,6,7]. Thus, novel materials and surface modifications are continuously developed to improve the clinical performance of dental abutments and implants [8,9]. 

In peri-implantitis, the degree of tissue damage is due to an unbalanced level of pro- and anti-inflammatory mediators, resulting in a dysregulated inflammatory response; the measure of the type and the quantity of the released cytokines is a good strategy for monitoring the condition of peri-implant tissues [10]. In periodontal inflammation, the neutrophils present in the gingival tissue are directed towards the bacteria of the dental plaque following specific chemotactic factors produced by the junctional epithelium (JE), the Intercellular Adhesion Molecule 1 (ICAM-1), necessary to help neutrophils in orienting themselves in molecular traffic [11]. Keratinocytes of healthy epithelium do not express ICAM-1, while keratinocyte ICAM-1 expression is detected in epithelial diseases: it localized in the inflammatory infiltration site of the epithelium and it is regulated by pro-inflammatory cytokines such as tumor necrosis factor-alpha (TNF-α) [12,13]. Therefore, regulation of ICAM-1 expression and differences in ICAM-1 levels in JE might depend not only on bacteria-derived lipopolysaccharides, but also on local inflammatory factors [12]. TNF-α is one of the main culprits in the progression of the gingival inflammation and bone resorption, stimulating both the osteoclast activity and the release of Matrix Metalloproteinases (MMPs), which, in turn, catalyze the degradation of extracellular matrix, connective tissue and alveolar bone [14]. MMP-9, in particular, appears to be a regulatory factor in neutrophil migration across the basement membrane [15] and its activity is negatively regulated by the Tissue Inhibitor of Metalloproteinase-1 (TIMP-1) [16], that, when regularly expressed, inhibits inflammation and tissue damage in the wound area after healing abutment implantation [14,17]. Among the landscape of anti-inflammatory mediators, Interleukin-10 (IL-10) has been reported to have a more powerful anti-inflammatory activity, with a pivotal role in the modulation of the inflammatory cascade in peri-implant tissues [10]. Interleukin 10 is able to inhibit the synthesis of pro-inflammatory cytokines, including TNFα. When IL-10 levels decrease, TNF-α levels are not regulated effectively anymore and rise, resulting in inflammation [18,19]. The expression of specific structural proteins mirrors the healthy status of the epithelium: cytokeratins (CKs) are a family of intermediate filament proteins of cytoskeleton and are the major structural proteins in epithelial cells [20]. The expression of different CKs characterizes the various differentiation stages of the epithelial cells, including oral mucosa [21]. Moreover, CK expression is modified by stress, apoptosis, malignant transformation, and other specific situations; in particular, CK-18 and CK-19 are not usually present in healthy oral gingival epithelium, but their presence characterizes malignant lesions; they are also detected during pocket formation due to the hyperproliferation typical of the chronic inflammation [22]. On the other hand, Desmoglein 3 (DSG-3), belongs to the desmosomal cadherins family and plays a role in the formation of desmosomes that join cells to one another. It is the predominant isoform in the oral mucosa and the decrease in its expression has been correlated with a weakening of the epithelial barrier against pathogenic organisms following the continuation of chemical, physical and inflammatory stimulation [23].

The extent of the inflammatory response and cell behavior are influenced by the material’s properties [24,25]. Consequently, increased attention is being paid to material characteristics such as surface chemistry, surface topography, micro-grooved surfaces, abutment macro and micro-designs [26], wettability [27] and the presence of biologically active proteins [28,29,30,31], which are all thought to influence the attachment of the JE and connective tissue to the abutment surface [24]. Implants are generally divided into main groups on the bases of their microstructural surface in “smooth” (or “machined”) and “rough”, whose surface irregularity enhances the bone integration; in addition to its roughness, the micromorphology of the surface also seems to influence the osteoblastic response to the implant, as bone-forming cells seem to have a particular affinity for titanium surfaces with a regular and uniform roughness [32,33]. 

In particular, laser machining is a selective, rapid and versatile process that does not alter the bulk properties of the materials. It produces micron and nanoscale patterns by melting and vaporizing the surface of the targeted material to generate micron and nanoscale patterns without altering the bulk properties of the material. Recently, a laser-treated technique Synthegra has been introduced to produce an implant topography characterized by nanopores with a diameter of 5 microns, an interpore distance of 15 microns, and a depth of 5 microns. Some studies have analyzed the influence of the surface topography on cell adhesion and bone integration, but no data are available on the inflammatory microenvironment generated by nanoscale laser-treated surface healing abutments. 

The aim of this study was to evaluate the inflammatory response induced in the soft tissues by a nanopored laser-treated surface when compared to a classic machined surface healing abutment. For this reason, we use a healing abutment whose surface presents alternated different treatments (machined/nanopored/machine/nanopored); this system allowed us to analyze the different responses evoked by the surface topography in the same implant. The expression of specific inflammatory markers by the soft tissues and the health status of the epithelial cells facing the two different (machined and laser-treated) surfaces were then analyzed. 

## 2. Results

**Figure 1:**
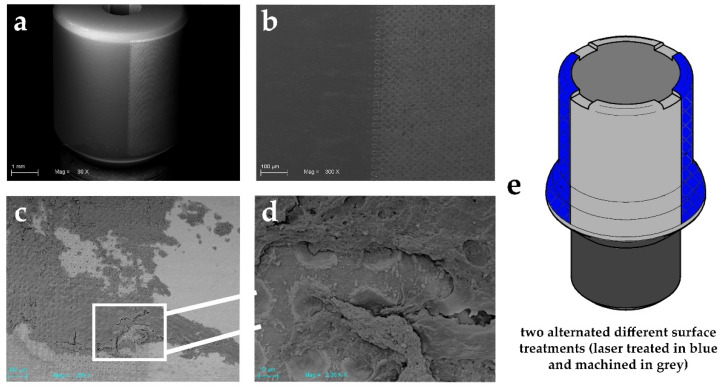
SEM images showing an experimental healing abutment used in this study: (**a**) showing the two different alternated surface treatments (laser treated on right and machined on left). Scale bar: 1 mm; (**b**) high magnification of these two surfaces topography. Scale bar: 100 μm; (**c**) soft tissue adhesion on both the studied surfaces. Please note the high quantity on the laser-treated surface compared to the machined one Scale bar: 100 μm; (**d**) higher magnification of the selected zone (Scale bar: 10 μm) and its interaction with soft tissue after biopsy; (**e**) technical design of experimental healing abutment.

In this study, we used an experimental healing abutment characterized by alternated nanopored laser-treated and machined surfaces (Figure 1). This particular design allowed us to analyze the response of the same subject to the two diverse surfaces, avoiding the bias of the different allocations among patients. The scanning electron microscopy performed on the gingival sample evidenced a more important amount of soft tissue around the laser-treated surface of the healing abutment compared to the machined one. 

**Figure 2:**
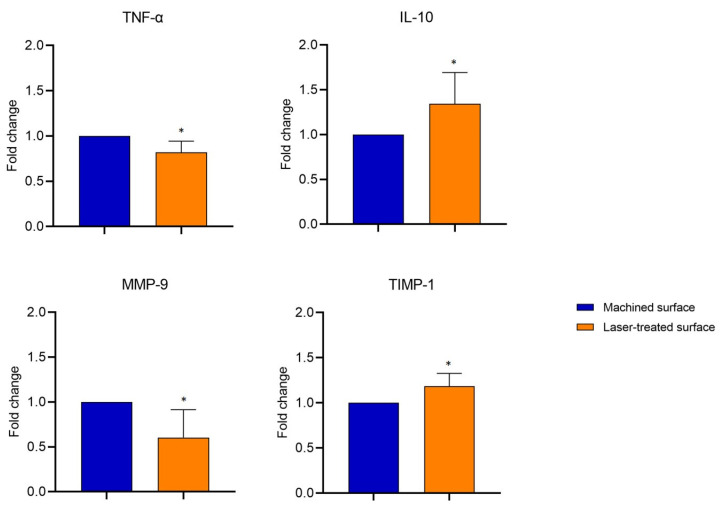
mRNA expression of *MMP-9, TIMP1*, *TNF-α,* and *IL-10* in soft tissue facing the laser-treated or machined surface by qPCR. Fold changes were determined from the -ΔΔCt values, calculated using *18S* as reference gene and normalized to the machined-surface, as the control condition. Values are expressed as mean ± SD of 15 different specimens.* indicates values statistically different (*p* < 0.05) between machined- and laser-treated surfaces.

The observation that epithelial cells better adhered to the nanoscale laser-treated surface rather than on the machined one prompted us to evaluate whether the different morphology of the healing abutment could also affect the health status of the soft tissue surrounding the surfaces. Therefore, first, we investigated the transcription profile of some inflammatory markers in the gingival biopsies facing the nanoscale laser-treated or machined surface. The qPCR analysis evidenced that gingival tissue around the laser-treated surface was characterized by significantly lower levels of TNF-α (−20%) and MMP-9 (−40%) and by higher levels of the TIMP-1 (−20%) (MMP-9 inhibitor) and IL-10 (−13%) mRNA, compared to the one facing the machined surface (Figure 2).

**Figure 3:**
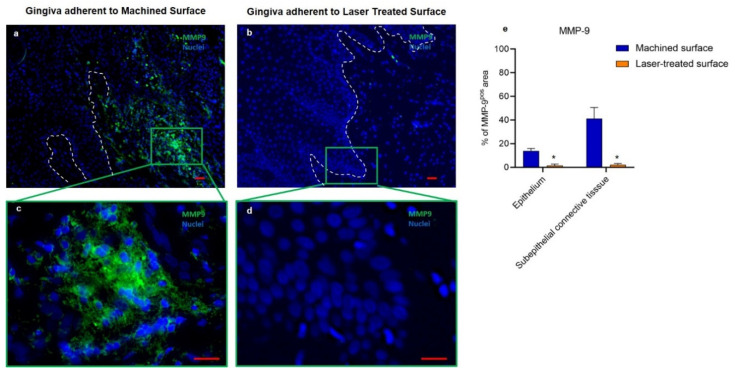
Immunofluorescence against MMP-9 (green) of the region of the gingiva adherent to the machined (**a**,**c**) or laser-treated (**b**,**d**) surfaces. The white dotted line indicates the basal lamina area that is regular in (**b**) and disrupted in (**a**). Nuclei are counterstained with DAPI. Scale bar: 10 µm. Images are representative of 15 different experiments (**e**) Percentage of ICAM-1 immunostaining positive area in the epithelial layers and the subepithelial connective tissue. Values represent the % of positive area, calculated as the mean of five different determinations in randomly chosen sections for each specimen. Values are expressed as mean ± SD * indicates values statistically different (*p* < 0.001) between the region of the gingiva adherent to the laser-treated- or to machined surfaces, as indicated.

As shown in Figure 3, the immunofluorescent analysis confirmed that MMP-9 was nearly undetectable around the laser-treated surface, while the immunostaining was evident in the epithelium and the connective tissue of the gingiva adherent to machined surfaces. Moreover, in MMP-9-positive areas, marked alterations and discontinuity of the basal lamina were also detected (Figure 3a,c). These observations suggest that the machine-treated surface of healing abutment induces a more intense local inflammatory response with MMP-9 release that alters the epithelial barrier. 

**Figure 4:**
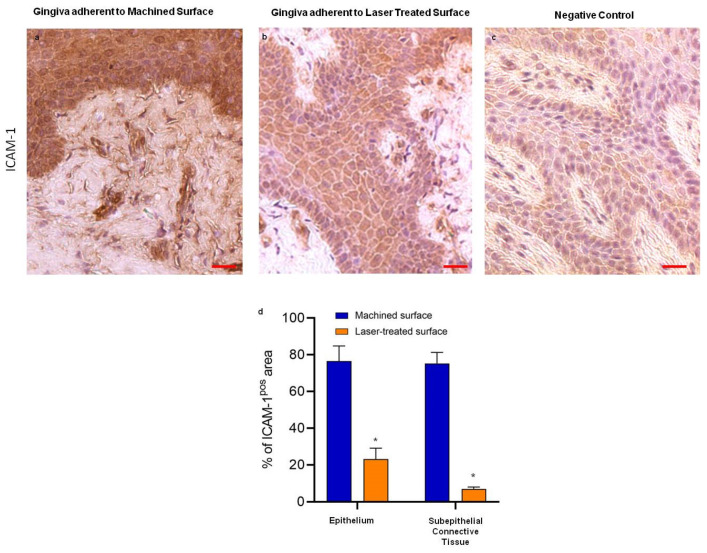
Immunostaining against ICAM-1 of the region of the gingiva adherent to the machined (**a**) and to laser-treated (**b**) surfaces, as indicated. (**c**) represents the negative control. Scale bar: 10 µm. Images are representative of 15 different experiments (**d**) Percentage of ICAM-1 immunostaining positive area in the epithelial layers and subepithelial connective tissue. Values are expressed as mean ± SD of five determinations in randomly chosen sections for each specimen. * indicates values statistically different (*p* < 0.001) between the region of the gingiva adherent to the laser-treated or to machined surfaces, as indicated.

Regarding the expression levels and the localization of the adhesion molecule ICAM-1, the immunohistochemical analysis evidenced that this chemoattractant molecule was only slightly expressed by the epithelium adherent to laser-treated surface, while it was dramatically overexpressed in the gingiva faced to machined one. Moreover, this positivity was accompanied by an evident morphological alteration at both epithelium and connective tissue levels (Figure 4).

**Figure 5:**
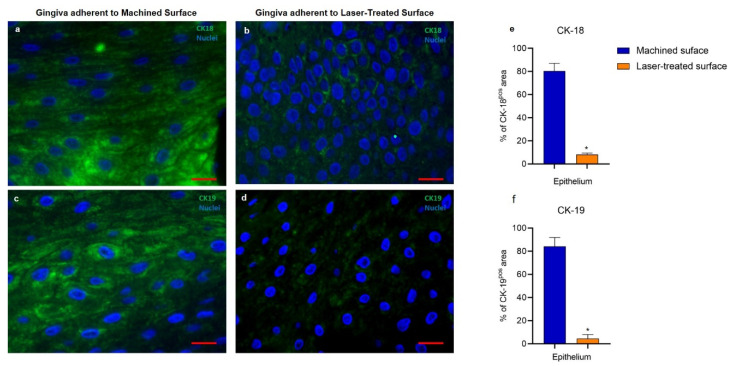
Immunofluorescence against CK-18 (green) (**a** and **b**) or CK-19 (green) (**c** and **d**) of the region of the gingiva adherent to the machined (**a**,**c**) or laser-treated (**b**,**d**) surface. Nuclei are counterstained with DAPI. Images are representative of 15 different experiments. Scale bar: 10 μm. Bar graphs show the percentage of CK-18 (**e**) and CK-19 (**f**) immunostaining positive areas in the epithelial layers. Values are expressed as mean ± SD of five determinations in randomly chosen sections for each specimen. * indicates values statistically different (*p* < 0.001) between the region of the gingiva adherent to the laser-treated- or to machined surfaces, as indicated.

Since alterations in the mucosal health often determine modification of the cytokeratins, the expression and distribution of CK-18 e CK-19 were investigated. These CK isotypes, normally absent in healthy epithelial cells, were strongly detected in the epithelium facing the machined surface. This evidence is in line with Chen et al. [34], who demonstrated that CK19 antigen was significantly increased in the oral epithelium of patients and was highly associated with the periodontal pocket formation. By contrast, the soft tissue in contact with the laser-treated surface showed a statistically significant weaker expression of both markers, indicating a less threatening effect of the micropored surface. (Figure 5).

**Figure 6:**
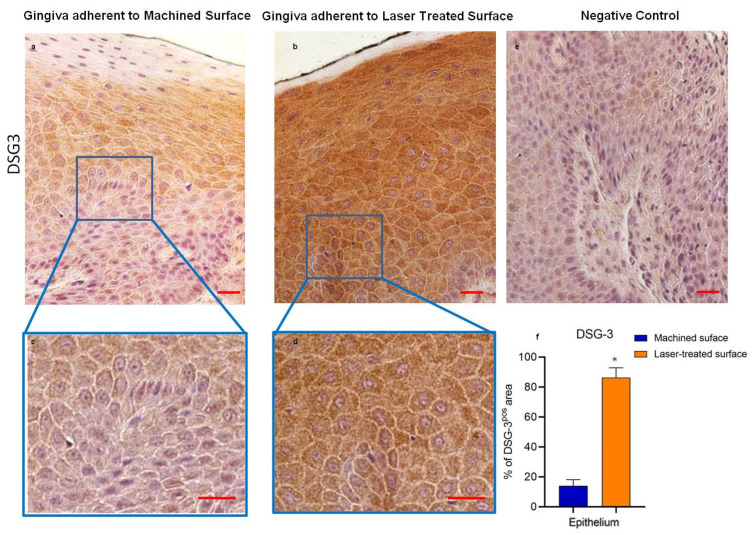
Immunostaining against DSG-3 of the region of gingiva adherent to the machined surface (**a**,**c**) or the laser-treated surface (**b**,**d**), as indicated. (**e**) represents the negative control. Images are representative of 15 different experiments. Scale bar: 10 µm. (**f**) Bar graphs show the percentage of DSG-3 immunostaining positive areas in the epithelial layers, as indicated. Values are expressed as mean ± SD of five determinations in randomly chosen sections for each specimen. * indicates values statistically different (*p* <0.001) between the region of the gingiva adherent to the laser-treated or to machined surfaces, as indicated.

To further analyze the effect of the different surfaces of the healing abutment on the cell architecture and tissue integrity, we analyzed the presence of DGS-3, whose activity is crucial for the formation of desmosomes in the oral mucosa. The immunohistochemical analysis evidenced that DGS-3 expression was significantly reduced in the epithelium facing the machined surface (Figure 6).

Table 1 summarized the expression of inflammatory markers and cytokeratin expression in soft tissues facing machined or nanopored laser-treated healing abutment.

## 3. Discussion

To evaluate whether the surface micromorphology of the healing abutment could affect the health of the soft tissue that faces it, we used a single healing abutment provided by alternated, nanopored laser-treated and machined surfaces, and investigated the expression of inflammatory markers in the gingival biopsies facing the different surfaces.

The present results suggest that the mucosal microenvironment around the laser-treated surface was characterized by a lower grade of inflammation than the soft tissue facing the machined-surface. This difference is effectively due to the diverse nano-topography of the implant surfaces, as cell behavior and inflammatory response can be tuned by the properties of the biomaterial. It is well known that the surface treatment and the different roughness aiming to increase the surface area available for osseointegration can influence the host response to the implant. Previous data on the fibrin clot on three different titanium topographies have demonstrated that the surface microtexture complexity influences the formation and the extension of the fibrin scaffold [25]. This is an important step for osseointegration and the early formation of an effective connective tissue seal. Indeed, it has been reported that, soon after the placement, the implant can absorb various molecules from the bloodstream in relation to the different characteristics of its surface, consequently leading to diverse tissue responses [35]. Moreover, the extracellular matrix adhesion proteins such as vitronectin and fibronectin can differently interact with the biomaterial surface, affecting the cellular adhesion, proliferation, and differentiation [36]. These differences in the cellular behaviors also determine a diverse inflammatory response. Previous studies performed on the effect of surface topography on leukocyte reactions showed that the surface roughness can directly affect the levels of the inflammatory cytokine released in the tissues surrounding the implant [37], thus affecting the activity of MMP-9 and the architecture of the tissue [38,39]. Moreover, the presence of nanopores on the titanium surface seems to promote the shift in the monocytes to the M2 phenotype, characterized by anti-fibrotic and anti-inflammatory properties [35]. Our data are in line with all these observations as the gingival tissue around the rough and porous laser-treated surface maintained a healthy architecture and expressed lower levels of pro-inflammatory cytokines and MMP-9. We can hypothesize that the porosity and the roughness of the laser-treated surface facilitate the absorption of extracellular matrix proteins that, activating ERK/MAPK and the TGF-β pathways [38,39], reduces the overall local inflammatory response. Further studies are needed to clarify the molecular mechanisms through which the nanopored laser surface affects the epithelial and inflammatory response to the healing abutment. 

It has been already reported that the upregulation of MMP-9, a regulatory factor in neutrophil migration across the basement membrane, is linked to the irreversible destruction of peri-implant tissue [14] and that low levels of TIMP-1 are associated with peri-implant disease progression [14]. Indeed, in healthy conditions, MMP-9 and TIMP-1 are in balance, but in many diseases and inflammatory processes, MMP-9 levels become higher, without a concomitant increase in TIMP-1. This unbalanced condition leads to tissue damage [40]. TIMP-1, in particular, is barely expressed in periodontitis in comparison with healthy subjects; on the other hand, its overproduction contributes to inhibiting the inflammation and tissue damage, due to the hyperactivation of MMP-9 [16], in the wound area after healing abutment implantation, suggesting that it exerts a pivotal role in controlling peri-implant disease progression [14,17]. The important role of MMP-9 in inflammation entails strict control of its expression. The analysis by qPCR of the fold change mRNA MMP-9 levels evidenced that gingiva facing the laser-treated surface of healing abutment expresses significantly less mRNA MMP-9 (about 40%). On the other hand, the immunohistochemical analysis of the mucosal MMP-9 protein expression showed a very important disparities in the mucosa facing the two different surfaces. The apparent discrepancy in the differences registered for MMP-9 gene expression with qPCR and MMP-9 protein expression by immunohistochemical analysis suggests that the translation of this inflammatory marker is also regulated at the posttranscriptional level. It is known, indeed, that even after a gene has been transcribed, its expression can still be regulated at various stages, and several mechanisms can modify the mRNA translation and its lifetime in the cytosol determining important modifications in protein expression; how the diverse nano-topography of the healing abutment may affect the post-transcriptional regulation of MMP-9 gene expression and its protein translation pathways needs more investigations. 

ICAM-1 expression by keratinocytes, fibroblasts, and endothelial cells is induced by inflammatory cytokines including TNF-α. Modification of its expression reflects local differences in cell populations and their state of activation [41]. In soft tissue facing the machined surface, we observed an increased expression in TNF-α, a proteases activator that induces fibroblasts apoptosis and osteoclasts proliferation, leading, consequently, to bone resorption together with reduced repair capacity of peri-implant tissues [14]. TNF-α, produced by macrophages, lymphocytes, and activated neutrophils [42] would induce the release of the neutrophil chemoattractant ICAM-1 and MMP-9, by establishing a self-maintenance loop that cannot be compensated for by the too-low levels of IL-10 and TIMP-1. On the other hand, in soft tissue facing the micropored laser-treated surface, high levels of TIMP-1 and IL-10 expressions might have a protective role [40,43,44], as already described that their levels in the peri-implant crevicular fluid negatively correlate with peri-implant disease [14]. TIMP-1 and IL-10 act, indeed, as anti-inflammatory molecules by downregulating MMP-9 and TNF-α and, in turn, reducing ICAM-1 release. 

The general inflammatory status that affect the gingival epithelium leads also to the increase in CK-18 and CK-19 expression, in line with previous studies that showed the overexpression of these two cytokeratins in junctional epithelium flogosi [45,46] and high positivity of CK19 in the epithelium inside the periodontal pocket and in the inflamed gingival epithelium [22]. Moreover, CK-19 expression is known to be directly proportional to the degree of proliferative activity and inversely proportional to E-cadherin [47,48]: accordingly, it has already been reported that E-cadherin levels are dramatically downregulated in the epithelium surrounding the machined titanium surfaces [8]. 

The epithelial cells are linked to each other mainly by adherens junctions and desmosomes that play a pivotal role in the maintenance of cell architecture and tissue integrity. These intercellular junctions are important in several physiological processes, including wound healing, cell polarization, and tissue morphogenesis; alterations in these junctions determine a failure of tissue homeostasis, leading to a variety of diseases. DSG-3 represents a fundamental component of desmosomes in the oral mucosa. The presence of this molecule represents a signal of forced adaptation of the oral epithelium to a physical stimulus, and supports the hypothesis of the crucial role of desmosomal cadherins in mediating communication between adjacent keratinocytes and in responding to external stimuli to which the oral mucosa can be exposed [49,50]. In this study, we observed that gingiva facing the machined surface of healing abutment is characterized by a lower expression of DSG-3. Based on these findings, we may hypothesize that the inflammatory microenvironment generated in the soft tissues by the machined surface affects the epithelial cells, reducing desmosome proteins and compromising cell–cell adhesion. 

Recently, it has been reported through histological findings that the gingiva adherent to this new laser-treated surface keeps a regular morphology in all its parts, while the region of the gingiva adherent to the machined surfaces shows several stress hallmarks, including highly inflamed areas with neutrophilic infiltration [8]. The authors concluded that the use of laser-treated surface on healing abutment could positively influence the healing of peri-implant soft tissues and it is preferable to maintain the integrity and functionality of the gingiva epithelium. On the contrary, some authors believe that the machined surface of the transgingival portion may influence the adhesion of bacteria, reducing it. Consequently, this kind of surface may reduce the risk of peri-implant soft and hard tissue inflammation [51,52]. A recent systematic review evidenced that material topography does not have an important impact on soft tissues around dental implants, contending that the abutment material may play a key role in the inflammatory grade [53]. However, the high heterogeneity on the effects of the abutment characteristics on peri-implant soft tissue health was reported (up to I2 > 90% when materials different from titanium were evaluated [53]). The findings of Sanz-Martin and et al. seem to contradict the traditional hypothesis that an increased surface roughness would facilitate biofilm formation, and therefore could have a negative influence on clinical periodontal parameters [54,55]. Thus, a turned transgingival collar should probably reduce the frequency of peri-implantitis; but the latter remains an enigmatic pathology with multifactorial characteristics [56].

Our results seem to encourage the hypothesis that the peri-implant soft tissue adhesion can be enhanced by surfaces with a moderate roughness. In fact, during the healing period after the second stage surgery, a rough-surfaced abutment could be advantageous for soft tissue integration. An SEM images also confirmed the results and encouraged the use of this nanoscale surface treatment. A greater attractiveness of the surface to the cells was demonstrated, in line with the literature, that showed the formation of the fibrin clot and a positive osteoblasts engraft on the surface [25,57]. Thus, the attractive capacity of this surface, combined with the mucosal reduced inflammatory state, encouraged the use of this surface in direct contact with soft tissues. The cellular adhesion obtained discourages the repeated screwing and unscrewing of the components which could damage the implant–abutment interface [58].

In conclusion, these results strongly support the hypothesis that the laser-treated surface of healing abutments generated a reduced inflammatory response in comparison to the machined one and preserve the healthy status of the epithelial cells. Thus, the use of the laser-treated surface for healing abutments in the clinical practice may reduce the risk of peri-implant soft tissue inflammation.

From a clinical point of view, these results, if maintained over time, can be of key importance in surface treatment of the transmucosal portion of the prosthetic restoration.

Study limitation: Although this study presents enthusiastic results for the role of this new laser-treated surface in preventing the inflammation of the soft tissues around healing abutments, it has a few limitations. It lacks a microbiological study of the bacteria film on the two different surfaces. Thus, additional microbiological studies on the presence of bacteria on the healing abutment surfaces are required and necessary. Another limit of the study is the short term of evaluation (only 3 months after implant placement) due to the difficulty of having biopsies once the permanent prosthesis is placed. 

## 4. Materials and Methods 

### 4.1. Patient Selection 

This study is part of a clinical trial approved by the “G.d’Annunzio” Chieti-Pescara University Ethic Committee N° 22 of 18.10.2018. A total of 38 patients, 25 men and 13 women (mean age 56.5 ± 9.9 years), were enrolled in this trial, but only 30 were included in the study. The other eight patients were excluded from the study as the keratinized tissue was not sufficient, thus not causing damage to the peri-implant soft tissues. The patients needed an implant-supported rehabilitation and were enrolled before implant placement. All patients come from the Implantology Operative Unit of the Department of Medical, Oral and Biotechnological Sciences, University “G.d’Annunzio” of Chieti-Pescara. They signed a written informed consent form and obtained information concerning the study protocol. Moreover, this was carried out following the principles set out in the Helsinki Declaration developed by the World Medical Association on Humans. Patients were enrolled following the inclusion and exclusion criteria. Briefly, patients between 18 and 75 years in good systemic and oral health and with an adequate bone and soft tissue quantity, were included in this study. On the contrary, patients with poor oral hygiene or active periodontal disease (Plaque Score (PS) and Bleeding on Probing (BOP) more than 25% at every stage), uncontrolled diabetes mellitus and smoking more than 10 cigarettes/day were excluded.

### 4.2. Surgical Treatment

The healing abutments are made of TiVaAl alloy, as most of the healing abutments were used during the clinical practice. This is made of grade 5; meanwhile, the implant fixture is made of commercially pure Titanium grade 4. 

All patients underwent a full clinical and radiographic oral evaluation. This was performed through panoramic and or computerized topographies. The implant insertion was achieved by a single surgeon (M.P) with local anesthesia with a 4% articaine solution containing 1:100,000 adrenaline. To uncover the bone profile, a total thickness flap was made. Then, the implant bed was prepared through the use of an increasing sequence of spiral drills under abundant irrigation. The implant positions were distributed as follows: 5 anterior maxilla, 14 posterior maxilla, 2 anterior mandible, 9 posterior mandible. All the implants inserted through a two-stage protocol were (Omny, Geass s.r.l, Pozzuoli del Friuli, Udine, Italy). The dimensions of the implants were from 7.0 to 11.5 mm in length, and 3.50 to 4.1 mm in diameter. A prophylaxis therapy was given to all the included patients (2 g/day for six days, Augmentin^®^, GLaxo-Smithkline Beecham, Brentford, UK). Then, the patients were instructed to wash their teeth carefully and use 0.20% chlorhexidine mouth rinses (Chlorexidine^®^, Oral-B, Boston, Waltham, MA, USA). The sutures were removed after 7–10 days.

The re-entry was done after a healing period 12 ± 4 weeks (T1) where all the HAs were placed. Each healing abutment had repeated the two surfaces as twice follows: laser-treated/machined/laser-treated/machined. This design was done to avoid the bias of the different allocation between patients. Experimental healing abutment was removed after 30 ± 7 days, the same time as the soft tissue biopsy. 

### 4.3. Specimen Retrieval and Analyses

After the healing period (30 ± 7 days), before starting with the prosthetic restoration of the fixtures, an impression was taken of the small-diameter Has, and circular sections (diameter 5 mm) soft tissues around them were retrieved for analysis as previously reported [8]. A total of 30 samples were retrieved: 15 of them were treated for quantitative real time PCR (qPCR), the other 15 were treated for immunohistochemical analysis.

### 4.4. RNA Extraction and Reverse Transcription

Total RNA from gingival tissue biopsies was extracted using QIAzol lysis reagent (QIAGEN, Hilden, Germany), according to the manufacturer’s procedure. The concentration of RNA was quantified by Qubit 3 (Thermo Fisher Scientific, Waltham, MA, USA). For reverse transcription, 1 μg of RNA was retro-transcribed by the High-Capacity cDNA reverse transcription kit (Thermo Fisher Scientific, Waltham, MA, USA) according to the manufacturer’s procedure.

### 4.5. Quantitative Real Time PCR (qPCR)

For all the examined mRNAs, qPCR analysis was performed using SYBR green (PowerUp SYBR Green Master mix, Thermo Fisher Scientific, Waltham, MA, USA) in the QuantStudio 3 (Thermo Fisher Scientific, Waltham, MA, USA). The run method consisted of the following steps: 95 °C for 10 min, 95 °C for 15 sec, 60 °C for 1 min. Steps 2 and 3 were repeated for 40 cycles. The authenticity of the PCR products was verified by melt–curve analysis. Each gene expression value was normalized to the expression level of *18S*. The fold changes were obtained by ΔΔCt methods, using the machined surface of the healing abutment as a control condition. 

The sequences of human primers were designed using NCBI Primer-Blast software and are listed in Table 2.

### 4.6. Immunohistochemical Analyses

The gingiva samples were fixed in 10% neutral buffered formalin and then embedded to paraffin, identifying the point of passage between the region of the gingiva adherent to the laser-conditioned and the one adherent to the machined surfaces. Three-µm-sized paraffin-embedded tissue sections, were deparaffinized, washed and blocked and then subjected to antigen retrieval, using 10 mM sodium citrate for 20 min at 60 °C. 

Immunohistochemistry was performed using the Mouse- and Rabbit-specific HRP/DAB (ABC) detection IHC kit (Abcam, Cambridge, UK) following the manufacturer’s instructions. Briefly, samples were then incubated with anti-human ICAM-1 or DSG-3 antibodies for 1 h. Successively, slides were incubated with goat anti-polyvalent antibody for 10 min, and subsequently with peroxidase for 10 min. After incubation, samples were washed and treated by DAB substrate. At the end of the process, slides were counterstained with hematoxylin-eosin [8]. Immunofluorescence staining was performed by blocking aspecificity and retrieving the antigen first, and incubating then the section with the primary anti-human Keratin 18 (2 µg/mL), or anti-human Keratin 19 (1:100) or anti-human MMP9 (1:200) antibodies overnight at 4 °C, as previously reported [60]. The samples were subsequently incubated with proper Alexa Fluor 488 goat anti-mouse or anti-rabbit secondary antibodies for 45 min. Nuclei were counterstained with DAPI (VECTASHIELD^®^ Vibrance™, Vector Laboratories, Burlingame, CA, USA). All the IHC and IF negative controls were performed in the absence of the primary antibodies. All the primary antibodies were purchased from ThermoFisher Scientific, USA. Histological observations were carried out using a Zeiss Axioskope light microscope (Zeiss, Jena, Germany) equipped with a Coolsnap Videocamera and the acquired images were analyzed with the MetaMorph 6.1 Software (Universal Imaging Corp, Downingtown, PA). Levels of primary antibody immunostaining were determined by analyzing the thresholded area percentage with the MethaMorph 6.1 program. Five randomly non-overlapping areas were chosen per each section, for the analysis as previously reported [61].

### 4.7. Scanning Electron Microscopy Analysis 

A Scanning Electron Microscopy (Carl Zeiss Evo 50, Oberkochen, Germany) was used to analyse the topography of the retrieved sHA as previously described [62]. The healing abutments were metallized through a gold-sputter Emitech K550 (Emitech Ltd., Ashford, UK) and subsequently inserted into the sample-holder for SEM analysis. The obtained images were then analysed using ImageJ software 1.48f 3D (Wayne Rasband, National Institutes of Health NIH, 375 Bethesda, Rockville, MD, USA) to identify the soft-tissue cells on both the surfaces. 

### 4.8. Statistical Analysis

A paired t-test using GraphPad Prism 4.0 was used to perform the statistical analysis between the two groups [63]. All data are presented as mean ± standard deviation (SD); a value of *p* < 0.05 was considered statistically significant.

## Figures and Tables

**Table 1:** Evaluation of inflammatory markers and cytokeratins expression in soft tissues facing machined or nanopored laser-treated healing abutment.

	Epithelium		Subepithelial Connective Tissue	
	Machined-Surface	Laser Treated Surface	Machined-Surface	Laser Treated Surface
Inflammatory Markers				
MMP-9	13.8 ± 2.1	1.4 ± 1.1 *	41.1 ± 9.2	2.3 ± 1.1 *
ICAM-1	76.8 ± 8.1	23.1 ± 5.9 *	75.1 ± 6.3	7.0 ± 1.2 *
Structural Proteins of keratinocytes				
CK-18	80.3 ± 6.5	8.1 ± 1.3 *	-	-
CK-19	84.1 ± 8.3	4.6 ± 3.4 *	-	-
DSG-3	14.2 ± 4.2	86.7 ± 6.1 *		

Values represent the % of positive in immunohistochemical analysis, calculated as the mean of five different determinations in randomly chosen sections for each specimen. Values are expressed as mean ± SD. * indicates statistical difference (*p* < 0.001) between the region of the gingiva adherent to the laser-treated vs. machined- surfaces.

**Table 2:** Primer sequences.

Gene	Sequence (5’ to 3’)
*MMP9* FW	CGCAGACATCGTCATCCAGT
*MMP9* RW	GGACCACAACTCGTCATCGT
*TIMP1* FW	CTGTTGGCTGTGAGGAATGC
*TIMP1* RW	CGGGACTGGAAGCCCTTTTC
*IL-10* FW	GTGAAAACAAGAGCAAGGCCG
*IL-10* RW	GCCACCCTGATGTCTCAGTTT
*TNF-α* FW	ATGAGCACTGAAAGCATGATCC
*TNF-α* RW	GAGGGCTGATTAGAGAGAGGTC
*18S* FW [59]	CATGGCCGTTCTTAGTTGGT
*18S* RW [59]	CGCTGAGCCAGTCAGTGTAG
